# From Laboratory to Field: *OsNRAMP5*-Knockdown Rice Is a Promising Candidate for Cd Phytoremediation in Paddy Fields

**DOI:** 10.1371/journal.pone.0098816

**Published:** 2014-06-05

**Authors:** Ryuichi Takahashi, Yasuhiro Ishimaru, Hugo Shimo, Khurram Bashir, Takeshi Senoura, Kazuhiko Sugimoto, Kazuko Ono, Nobuo Suzui, Naoki Kawachi, Satomi Ishii, Yong-Gen Yin, Shu Fujimaki, Masahiro Yano, Naoko K. Nishizawa, Hiromi Nakanishi

**Affiliations:** 1 Graduate School of Agricultural and Life Sciences, The University of Tokyo, Bunkyo-ku, Tokyo, Japan; 2 Graduate School of Science, Tohoku University, Aoba-ku, Sendai, Miyagi, Japan; 3 Agrogenomics Research Center, National Institute of Agrobiological Sciences, Tsukuba, Ibaraki, Japan; 4 Quantum Beam Science Directorate, Japan Atomic Energy Agency, Takasaki, Gunma, Japan; 5 Research Institute for Bioresources and Biotechnology, Ishikawa Prefectural University, Nonoichi-shi, Ishikawa, Japan; Universidade Federal do Rio Grande do Sul, Brazil

## Abstract

Previously, we reported that OsNRAMP5 functions as a manganese, iron, and cadmium (Cd) transporter. The shoot Cd content in *OsNRAMP5* RNAi plants was higher than that in wild-type (WT) plants, whereas the total Cd content (roots plus shoots) was lower. For efficient Cd phytoremediation, we produced *OsNRAMP5* RNAi plants using the natural high Cd-accumulating cultivar Anjana Dhan (A5i). Using a positron-emitting tracer imaging system, we assessed the time-course of Cd absorption and accumulation in A5i plants. Enhanced ^107^Cd translocation from the roots to the shoots was observed in A5i plants. To evaluate the phytoremediation capability of A5i plants, we performed a field experiment in a Cd-contaminated paddy field. The biomass of the A5i plants was unchanged by the suppression of *OsNRAMP5* expression; the A5i plants accumulated twice as much Cd in their shoots as WT plants. Thus, A5i plants could be used for rapid Cd extraction and the efficient phytoremediation of Cd from paddy fields, leading to safer food production.

## Introduction

Cadmium (Cd) is a toxic heavy metal that causes serious health problems in humans. Cd, which was a well-known cause of ‘itai-itai’ in Japan in the past, was recently classified as a human carcinogen by the International Agency for Research on Cancer [1]. Cd accumulates in the human body through food, and the main source of dietary Cd intake among Asians is rice [2]–[4]. In rice plants at the grain-filling stage, Cd is absorbed directly by the roots, moves to the panicles, and accumulates in the grain [5], [6]. Therefore, reducing the Cd level in paddy field soil is necessary to ensure food safety.

Phytoremediation is an effective method for removing various soil contaminants using plants. Rice is a good candidate for Cd phytoremediation due to its large biomass and well-established cultivation and harvesting methods [7]. There is significant genotypic variation in the Cd levels of rice grains and shoots [8]–[10]; this said, the cultivar Anjana Dhan naturally accumulates more Cd in its grains and shoots than any other cultivar in the world [10]. Phytoremediation using high Cd-accumulating cultivars successfully reduced the total soil Cd content and subsequent grain Cd content [11], [12].

Natural resistance-associated macrophage proteins (NRAMPs) comprise a large family of membrane proteins that function as general metal ion transporters [13]–[20]. Cd is transported through essential metal transporters [21], and NRAMPs are thought to be a major route of Cd transport in plants. In *Arabidopsis*, AtNRAMP1, AtNRAMP3, and AtNRAMP4 have been reported to transport Cd in addition to iron (Fe) and manganese (Mn) [14], [22]–[26]. AtNRAMP6, a homolog of AtNRAMP1, does not transport Fe and Mn, but it functions in the intracellular distribution of Cd [27]. In rice, OsNRAMP1 participates in the uptake of Cd in addition to Fe [22], [28]. Furthermore, OsNRAMP5 (Os07g0257200), which is involved in the constitutive uptake of Cd in roots, is recognized as a major route of Cd entry into root cells [29], [30]. Previously, we reported that *OsNRAMP5*-knockdown plants accumulated increased amounts of Cd in their shoots, whereas the total Cd content (roots plus shoots) was reduced; thus, these plants showed potential for Cd phytoremediation [29].

The positron-emitting tracer imaging system (PETIS) is a radiotracer-based imaging method that enables real-time monitoring of the movement of a tracer in living plants and the quantitative analysis of that movement. Using this system, the translocation of Fe, zinc (Zn), and Mn was investigated in rice and barley [31]–[36]. Furthermore, the uptake and translocation of Cd was investigated in rice and oilseed rape using this system [5], [6], [37].

In this study, we examined the absorption and translocation of Cd in *OsNRAMP5*-knockdown rice plants using radioisotopes. Furthermore, we carried out a field experiment to evaluate the ability of these plants to extract Cd from paddy soil.

## Materials and Methods

### Plant Materials and Growth Conditions

Seeds of the *Oryza sativa* cultivar Anjana Dhan were germinated for 2 weeks on Murashige and Skoog (MS) medium at 28°C under a 16-h light/8-h dark photoperiod. *OsNRAMP5* RNAi (A5i) plants were constructed as described previously [29], and the transgenic rice seeds were germinated on MS medium containing 50 mg L^−1^ hygromycin B. After germination, the seedlings were transferred to a 20-L plastic container and grown in a greenhouse (30°C, natural light). The composition of the nutrient solution was as follows: 0.7 mM K_2_SO_4_, 0.1 mM KCl, 0.1 mM KH_2_PO_4_, 2.0 mM Ca (NO_3_)_2_, 0.5 mM MnSO_4_, 0.1 mM Fe (III)-EDTA, 10 µM H_3_BO_3_, 0.5 µM MnSO_4_, 0.5 µM ZnSO_4_, 0.2 µM CuSO_4_, and 0.05 µM Na_2_MoO_4_. The nutrient solution was adjusted to pH 5.5 with 1 M HCl every day and changed two times per week. For the Cd treatments, 3-week-old plants were transferred to a nutrient solution containing 0.1 µM CdCl_2_ and cultivated for 2 additional weeks. To compare the metal concentrations in the presence of Fe (II) or Fe (III), we added 10 µM FeSO_4_ instead of 0.1 mM Fe (III)-EDTA. The nutrient solution was adjusted to pH 5.5 with 1 M HCl every day and changed every 2 days.

Field experiments were established at the experimental paddy field of Gyeongsang National University, Gyongnam, Korea (35°02′N, 128°03′E). Cd was added artificially; its concentration was 0.43 mg Cd kg^–1^ dry weight of soil, as determined by extraction with 0.1 M HCl. Seeds were germinated as described previously, and the seedlings were transplanted to the paddy field [38], [39]. When the plants entered the heading stage, irrigation was stopped and drainage was maintained until harvesting.

### PETIS

^107^Cd was produced as described previously [5] at Takasaki Ion Accelerators for Advanced Radiation Application (Japan Atomic Energy Agency, Takasaki, Japan). ^107^Cd and nonradioactive Cd at a concentration of 0.1 µM were supplied simultaneously to the nutrient solution when imaging was started, and the Cd concentration was maintained at 0.1 µM during the experiments. Plants were placed between the detectors of the PETIS (a modified PPIS-4800; Hamamatsu Photonics, Hamamatsu, Japan) as described previously [5]. The radioactivity of ^107^Cd in the detected region was measured by region of interest (ROI) analysis, and the data obtained from the PETIS were reconstructed using ImageJ 1.42 software (http://rsb.info.nih.gov/ij). Each ROI was extracted from the data, and time courses of signal intensity were generated. The PETIS experiments were performed twice, each using two A5i plants and two wild-type (WT) plants (n = 4).

After the PETIS experiment, autographic images were obtained using a bio-imaging analyzer (BAS-1500; Fuji Film, Tokyo, Japan).

### Measurement of Plant Metal Concentrations

The plants were harvested and dried at 70°C for 2 days. Samples (80–150 mg) were then digested with 3 mL of 13 M HNO_3_ using MARS XPRESS (CEM, Tokyo, Japan). The digestion time and temperature were 30 min at 220°C for rice grown in hydroponic culture and 60 min at 220°C for rice grown in the field, respectively. The metal concentrations were measured using inductively coupled plasma-atomic emission spectrometry (SPS1200VR; Seiko, Tokyo, Japan). Three biological replicates were used for hydroponic culture and ten were used for the field experiment.

### Expression Analysis of OsNRAMP1 and OsIRT1

Total RNA was extracted from rice using an RNeasy Plant Mini Kit (Qiagen, Hilden, Germany). The RNA was reverse-transcribed (RT) using an oligo dT primer and ReverTra Ace Reverse Transcriptase (Toyobo, Tokyo, Japan). Quantitative RT-PCR was then performed using the Smart Cycler System (Takara, Shiga, Japan). Amplification of *OsNRAMP1* (Os07g0258400) and *OsIRT1* (Os03g0667500) was performed using primer pairs as described previously [28], [31] with SYBR Premix Ex Taq (Perfect Real Time; Takara). As an internal standard, *α-tubulin* (Os03g0726100) was used as described previously [31]. Transcript abundance was normalized to the *α-tubulin* expression level as ratios to *OsNRAMP1* and *OsIRT1*. The results represent the average numbers of transcripts in 1 µg of total RNA in three reactions.

## Results

### Analysis of ^107^Cd Transport Using a PETIS

**Figure 1:**
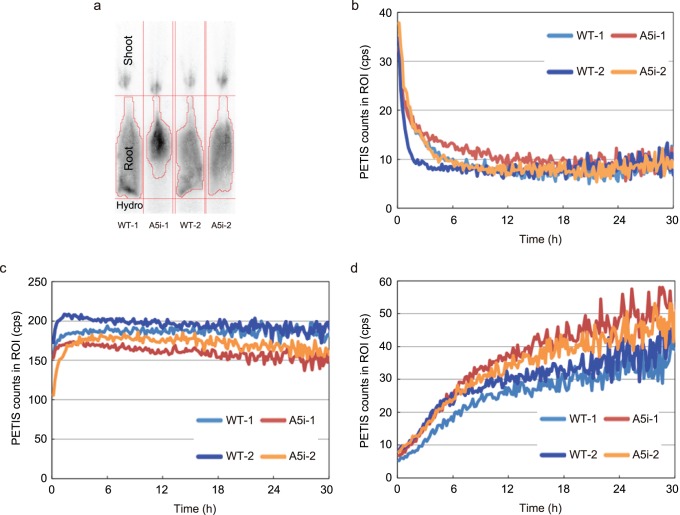
^107^Cd uptake and transport in Anjana Dhan *OsNRAMP5* RNAi plants. (A) Regions of interest were set and used to generate time-activity curves of the hydroponic solution (Hydro), roots, and shoots, respectively. (B–D) Time course of the Cd counts in the hydroponic solution (B), roots (C), and shoots (D).

After the addition of ^107^Cd to the nutrient solution, ^107^Cd absorption by the roots was observed immediately in both A5i and WT plants (Figure 1B and 1C). The amount of Cd in the roots increased within 1 h after exposure to ^107^Cd and subsequently decreased (Figure 1C). The amount of ^107^Cd was higher in the roots of the WT plants than in those of the A5i plants throughout the course of imaging. However, the ^107^Cd level was higher in the shoots of A5i plants than in those of WT plants (Figure 1D). Increased ^107^Cd accumulation was also observed in the leaves of A5i, as compared to WT plants, in the BAS images (Figure S1). We performed our analysis twice using a PETIS; similar results were obtained in both experiments (Figure S2).

### Metal Concentrations in Hydroponic Culture

**Figure 2:**
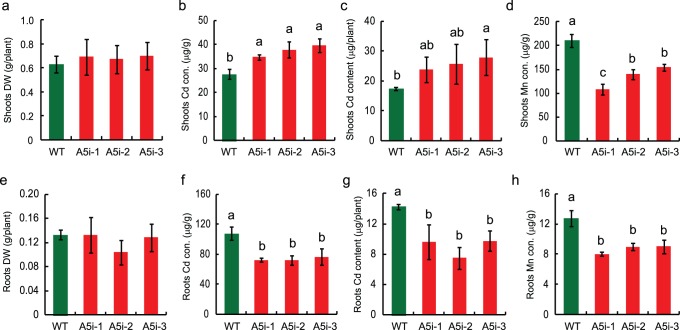
Metal concentrations in Anjana Dhan *OsNRAMP5* RNAi (A5i) plants. (A) Shoot dry weights of WT and A5i plants. (B, C) Cd concentration (B) and Cd content (C) in the shoots of A5i plants. (D) Mn concentration in the shoots of A5i plants. (E) Root dry weights of WT and A5i plants. (F, G) Cd concentration (F) and Cd content (G) in the roots of A5i plants. (H) Mn concentration in the roots of A5i plants. Plants were grown in the presence of 0.1 µM CdCl_2_ for 2 weeks. The results are presented as the means ± SD of three plants (n = 3). Different letters indicate significant differences at *P*<0.05 according to Duncan’s test.

When plants were grown in hydroponic solution, there was no significant difference in the dry weights of the shoots and roots between A5i and WT plants (Figures 2 and S3, in the presence and absence of Cd in the solution, respectively). In the presence of 0.1 µM Cd, the Cd concentration in the shoots of A5i plants was higher than in WT plants (Figure 2B). In the shoots of A5i-3 plants, the Cd content was 1.6-fold higher than that in WT plants (Figure 2C). On the other hand, the Cd concentration and Cd content in the roots of A5i plants were significantly lower than those in the roots of WT plants (Figure 2F and 2G). In the presence of 0.1 µM Cd, the total Cd content (roots plus shoots) in A5i plants was equal to that in WT plants. The Mn concentrations in the shoots and roots were lower than those in WT plants (Figure 2D and 2H). The shoot and root concentrations of other essential metals (Zn, Fe, and Cu) were almost equal in A5i and WT plants in the presence of Cd (Figure S4).

### Expression Analysis

**Figure 3:**
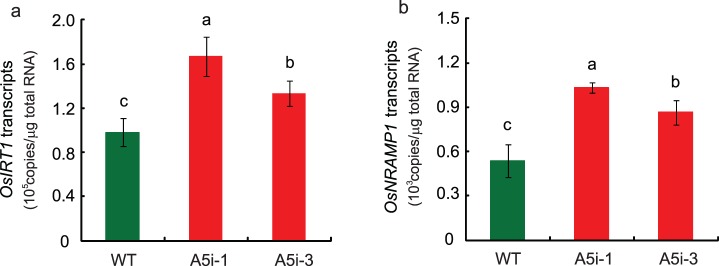
Expression analysis of Anjana Dhan *OsNRAMP5* RNAi (A5i) plants. Expression of *OsIRT1* (A) and *OsNRAMP1* (B) in the roots of A5i plants in the absence of Cd. The results are presented as the means ± SD of three reactions. Different letters indicate significant differences at *P*<0.05 according to Duncan’s test.

Cd uptake and translocation is mediated in part by Fe transporters such as OsIRT1, OsIRT2, and OsNRAMP1 [28], [31], [40], [41]. When plants were grown in normal nutrient solution, the expression of *OsIRT1* and *OsNRAMP1* was higher in the roots of A5i plants compared to WT plants (Figure 3).

### Field Experiments

**Figure 4:**
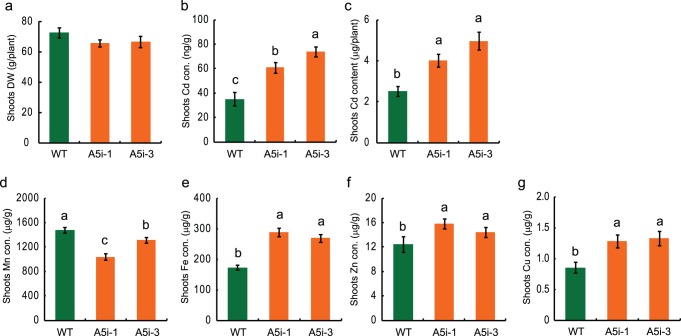
Field trial of Cd phytoremediation by Anjana Dhan *OsNRAMP5* RNAi (A5i) plants. (A) Shoot dry weights of WT and A5i plants. (B, C) Cd concentration (B) and Cd content (C) in the shoots of A5i plants. (D–G) Concentrations of Mn (D), Fe (E), Zn (F), and Cu (G) in the shoots of A5i plants. The plants were grown in a paddy field. The results are presented as the means ± SE (n = 10). Different letters indicate a significant difference from wild type at *P*<0.05 according to Duncan’s test.

When plants were grown in an isolated paddy field, the A5i plants showed normal growth (Figure S5) with no significant difference in shoot weight (Figure 4A). The Cd concentration and the Cd content in the shoots of A5i plants were higher than in those of WT plants (Figure 4B and 4C). The Cd concentration and Cd content in the shoots of A5i plants were up to 2.1- and 2.0-fold higher than in those of WT plants, respectively (Figure 4B and 4C). The shoot Mn concentration in A5i plants was lower than that in WT plants, whereas the concentrations of Zn, Fe, and Cu were higher than those in WT plants (Figure 4D–G).

## Discussion

Previously, we showed that OsNRAMP5 functions as a Cd, Fe, and Mn transporter in rice [29], [42]. *OsNRAMP5* is expressed mainly in roots, and the protein is localized to the plasma membrane [29]. Constitutive *OsNRAMP5* expression revealed that OsNRAMP5 may be a major transporter for Cd uptake [29], [30]. The significant decrease in ^107^Cd concentration in the roots of the A5i plants (Figures 1 and S2) also suggests that OsNRAMP5 is a major transporter for Cd uptake.

Using a PETIS, a higher level of ^107^Cd was observed in the shoots of A5i plants as compared to WT plants, whereas the amount of ^107^Cd in the roots of the A5i plants was lower than that in the roots of the WT plants (Figures 1 and S2). These results suggest that ^107^Cd translocation from roots to shoots was enhanced in the A5i plants. High Cd-accumulating cultivars are characterized by rapid and abundant Cd translocation from roots to shoots, as compared to low Cd-accumulating cultivars [6]. Cd transfer to the shoots of the A5i plants was found to be more rapid and more abundant than in Anjana Dhan, one of the highest Cd-accumulating cultivars, suggesting that A5i plants are promising candidates for practical Cd phytoremediation.

The Cd concentration and Cd content in the shoots of *OsNRAMP5*-knockdown plants were higher than those in WT plants (Figure 2). A higher shoot Cd concentration was reported in cultivar Tsukinohikari *OsNRAMP5* RNAi plants (T5i) [29]. Cd uptake and translocation is mediated in part by Fe transporters such as OsIRT1, OsIRT2, and OsNRAMP1 [28], [31], [40], [41]. The expression of *OsIRT1*, *OsIRT2*, and *OsNRAMP1* in T5i plants was higher than that in WT plants; thus, the induction of these transporters enhances Cd translocation to shoots [29]. The expression of *OsIRT1* and *OsNRAMP1* was also higher in the roots of A5i plants, as compared to WT plants (Figure 3). It is possible that increased expression of these genes enhanced Cd translocation from roots to shoots, resulting in the increased accumulation of Cd in the shoots of both A5i and T5i plants.

Previously, we performed hydroponic culture under 10 µM Cd [29]. Since Sasaki *et al*. [30] showed that high Cd accumulation in T5i and A5i shoots could be due to indirect effects, we investigated Cd accumulation under 0.1 µM Cd, which is the concentration used by Sasaki *et al*. [30]. Under 0.1 µM Cd, the total Cd content (roots plus shoots) of A5i plants was not reduced compared to WT plants (Figure 2). This was likely due to the lower Cd concentration used in this study. Nevertheless, a higher Cd concentration in the shoots was observed in both T5i and A5i plants not only at 10 µM Cd [29] but also at 0.1 µM Cd (Figure 2). In contrast, Sasaki *et al*. [30] reported that the shoot Cd concentration in *OsNRAMP5* RNAi plants was lower than that in WT plants. This contradiction might have been due to the difference in expression level of *OsNRAMP5*. As OsNRAMP5 is thought to be a major Cd uptake transporter from the soil, if the function of OsNRAMP5 was completely disrupted, the root and shoot Cd concentrations would be extremely low [43]. In our A5i plants, the expression of *OsNRAMP5* was 1/2 to 2/3 that in WT plants [29], whereas the expression of *OsNRAMP5* was extremely suppressed in the *OsNRAMP5* RNAi plants used by Sasaki *et al*. [30]. This result suggests that functional OsNRAMP5 was present in the A5i plants, and that A5i plants take up less Cd by OsNRAMP5 as well as OsIRT1, OsIRT2, and OsNRAMP1. OsNRAMP5 may also be involved in constitutive Fe uptake [42]. Sasaki *et al*. [30] used FeSO_4_ as an Fe source in their hydroponic culture solution, whereas we used Fe (III)-EDTA. In the presence of Fe (II), the root Fe concentration was much higher than in the presence of Fe (III) (Figure S6). The expression of some Fe-deficiency-inducible genes was not up-regulated under conditions of Fe (II) sufficiency [44], and the expression of *OsIRT1* and *OsNRAMP1* was induced only in the presence of Fe (III) (Figure S6). These results indicate that the expression of *OsIRT1* and *OsNRAMP1* was not induced due to Fe sufficiency in the roots in the presence of Fe (II). Moreover, the Cd concentration in the shoots of the A5i plants was higher in the presence of Fe (III), as compared to that in the presence of Fe (II) (Figure S6). These results clearly indicate that Cd translocation from roots to shoots was enhanced to a greater extent in the presence of Fe (III) compared to the presence of Fe (II). In practical phytoremediation using rice, the water in a paddy field is drained after the tilling stage to maximize Cd accumulation in the shoots [11], [12]. Under such oxidative conditions, Fe is oxidized and exists mainly as Fe (III) in the soil. Therefore, the uptake and translocation of Cd in A5i plants in the presence of Fe (III) would reflect the field conditions more than the use of Fe (II).

Phytoremediation over a 2-year period using one of the highest Cd-accumulating cultivars, Cho-ko-koku, reduced the total soil Cd content by 38%, as compared to the control, whereas phytoremediation over a 3-year period using the relatively high Cd-accumulating *indica* cultivars IR8 and Milyang 23 reduced the total soil Cd content by 20 and 23%, respectively [12]. Cd accumulation in the shoots of Anjana Dhan was equal to that in Cho-ko-koku [10]. In this study, we found that the 2.0-fold increase in Cd accumulation in the shoots resulted from the knockdown of *OsNRAMP5* in Anjana Dhan in a Cd-contaminated field (Figure 4), and the soil Cd concentration was reduced from 0.43 to 0.26 mg Cd kg^–1^ dry weight of soil after 1 year. Increased Cd accumulation in the shoots is available for phytoremediation in the field because only shoots are usually harvested for rice phytoremediation. The A5i plants accumulated considerably more Cd compared to the reported high-Cd-accumulating cultivars; thus, Cd phytoremediation using A5i plants will contribute to both the rapid and efficient extraction of Cd from paddy fields and safer food production.

## Supporting Information

Figure S1 **Autoradiography of the shoots following PETIS analysis.** (A) Photograph of WT and Anjana Dhan *OsNRAMP5* RNAi (A5i) plants. (B) BAS images of WT and A5i plants after sufficient decay of ^107^Cd in the plants.
(EPS)

Figure S2 **^107^Cd uptake and transport in Anjana Dhan** ***OsNRAMP5*** **RNAi plants in a second independent experiment.** (A) Regions of interest were set and used to generate time-activity curves of the hydroponic solution (Hydro), roots, and shoots, respectively. (B–D) Time course of the Cd counts in the hydroponic solution (B), roots (C), and shoots (D).
(EPS)

Figure S3 **Metal concentrations in** **Anjana Dhan** ***OsNRAMP5*** **RNAi (A5i) plants in the absence of Cd.** (A) Shoot dry weights of WT and A5i plants. (B–E) Concentrations of Zn (B), Mn (C), Fe (D), and Cu (E) in the shoots of A5i plants. (F) Root dry weights of WT and A5i plants. (G–J) Concentrations of Zn (G), Mn (H), Fe (I), and Cu (J) in the roots of A5i plants. Plants were grown in the absence of Cd for 2 weeks. The results are presented as the means ± SD (n = 3). Different letters indicate significant differences at *P*<0.05 according to Duncan’s test.
(EPS)

Figure S4 **Metal concentrations in** **Anjana Dhan** ***OsNRAMP5*** **RNAi (A5i) plants in the presence of Cd.** (A–C) Concentrations of Zn (A), Fe (B), and Cu (C) in the shoots of A5i plants. (D–F) Concentrations of Zn (D), Fe (E), and Cu (F) in the roots of A5i plants. The results are presented as the means ± SD (n = 3). Different letters indicate significant differences at *P*<0.05 according to Duncan’s test.
(EPS)

Figure S5 **Photographs of field-grown WT plants (A) and Anjana Dhan** ***OsNRAMP5*** **RNAi (A5i) plants (B).**
(EPS)

Figure S6 **Expression analysis and metal concentrations in Anjana Dhan** ***OsNRAMP5*** **RNAi (A5i) plants grown in the presence of Fe (II) or Fe (III).** *OsIRT1* (A) and *OsNRAMP1* (B) expression in the roots of A5i plants. (C) Shoot dry weights of WT and A5i plants. (D–H) Concentrations of Cd (D), Zn (E), Mn (F), Fe (G), and Cu (H) in the shoots of A5i plants. (I) Root dry weights of WT and A5i plants. (J–N) Concentrations of Cd (J), Zn (K), Mn (L), Fe (M), and Cu (N) in the roots of A5i plants. Different letters indicate significant differences at *P*<0.05 according to Duncan’s test. The results are presented as the means ± SD (n = 3).
(EPS)
